# Hexaaqua­zinc(II) bis­[tris­(3-carb­oxy­pyridine-2-carboxyl­ato)zincate(II)]

**DOI:** 10.1107/S1600536810047252

**Published:** 2010-11-20

**Authors:** Mehrnaz Gharagozlou, Vratislav Langer, Andya Nemati

**Affiliations:** a Department of Nanotechnology and Nanomaterials, Institute for Color Science and Technology, PO Box 16765-654 Tehran, Iran; b Environmental Inorganic Chemistry, Department of Chemical and Biological Engineering, Chalmers University of Technology, SE-412 96 Gothenburg, Sweden; c Iran Compiling Encyclopedia Foundation, Tajrish, Tehran, Iran

## Abstract

The title compound, [Zn(H_2_O)_6_][Zn(C_7_H_4_NO_4_)_3_]_2_, consists of two [Zn(py-2,3-dcH)_3_]^−^ anions (py-2,3-dcH is 3-carboxy­pyridine-2-carboxylate) and one [Zn(H_2_O)_6_]^2+^ cation. The anion is a six-coordinate complex located on a threefold rotation axis with a slightly distorted octa­hedral geometry around Zn^2+^ ion. The cation is also six-coordinate with an octa­hedral geometry around the Zn atom, located at a 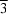 axis. Non-covalent inter­actions such as π–π stacking [centroid–centroid distance = 3.828 (4)Å] and O—H⋯O hydrogen bonds play important roles in stabilizing the supra­molecular structure.

## Related literature

For first-row transition metal complexes of pyridine-2,3-dicarb­oxy­lic acid and various bases and for Zn—O distances, see: Aghabozorg, Daneshvar *et al.* (2007 ▶); Aghabozorg, Sadr-khanlou *et al.* (2007) ▶; Goher *et al.* (1993 ▶); Kang *et al.* (2006 ▶); Li *et al.* (2006 ▶); Prior & Rosseinsky (2001 ▶); Swiegers & Malefetse (2000 ▶); Yin & Liu (2009 ▶). For hydrogen-bond motifs, see: Bernstein *et al.* (1995 ▶). 
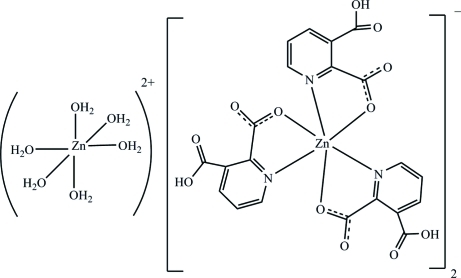


## Experimental

### 

#### Crystal data

[Zn(H_2_O)_6_][Zn(C_7_H_4_NO_4_)_3_]_2_
*M* *_r_* = 1300.88
Trigonal, 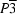

*a* = 14.470 (4) Å
*c* = 6.284 (2) Å
*V* = 1139.4 (6) Å^3^
*Z* = 1
Mo *K*α radiation
μ = 1.68 mm^−1^
*T* = 153 K
1.20 × 0.44 × 0.42 mm

#### Data collection

Siemens SMART CCD area-detector diffractometer
Absorption correction: multi-scan (*SADABS*; Sheldrick, 2003 ▶) *T*_min_ = 0.237, *T*_max_ = 0.538
10776 measured reflections
1356 independent reflections
1009 reflections with *I* > 2σ(*I*)
*R*_int_ = 0.154

#### Refinement

*R*[*F* ^2^ > 2σ(*F* ^2^)] = 0.061
*wR*(*F* ^2^) = 0.158
*S* = 1.00
1356 reflections
133 parameters
4 restraints
H atoms treated by a mixture of independent and constrained refinement
Δρ_max_ = 0.86 e Å^−3^
Δρ_min_ = −0.82 e Å^−3^

### 

Data collection: *SMART* (Bruker, 2003 ▶); cell refinement: *SAINT* (Bruker, 2003 ▶); data reduction: *SAINT*; program(s) used to solve structure: *SHELXS97* (Sheldrick, 2008 ▶); program(s) used to refine structure: *SHELXL97* (Sheldrick, 2008 ▶); molecular graphics: *DIAMOND* (Brandenburg, 2010 ▶); software used to prepare material for publication: *SHELXTL* (Sheldrick, 2008 ▶).

## Supplementary Material

Crystal structure: contains datablocks I, global. DOI: 10.1107/S1600536810047252/om2373sup1.cif

Structure factors: contains datablocks I. DOI: 10.1107/S1600536810047252/om2373Isup2.hkl

Additional supplementary materials:  crystallographic information; 3D view; checkCIF report

## Figures and Tables

**Table 1:** Selected bond lengths (Å)

Zn1—O1	2.083 (4)
Zn1—N1	2.157 (5)
Zn2—O5	2.095 (5)

**Table 2:** Hydrogen-bond geometry (Å, °)

*D*—H⋯*A*	*D*—H	H⋯*A*	*D*⋯*A*	*D*—H⋯*A*
O3—H3⋯O2^iv^	0.83 (5)	1.72 (2)	2.547 (5)	169 (7)
O5—H52⋯O4	0.83 (5)	2.38 (4)	3.157 (7)	156 (7)
O5—H51⋯O4^v^	0.82 (6)	2.02 (3)	2.795 (6)	158 (6)

Symmetry codes: (iv) 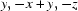; (v) 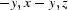.

**Fig. 1.**
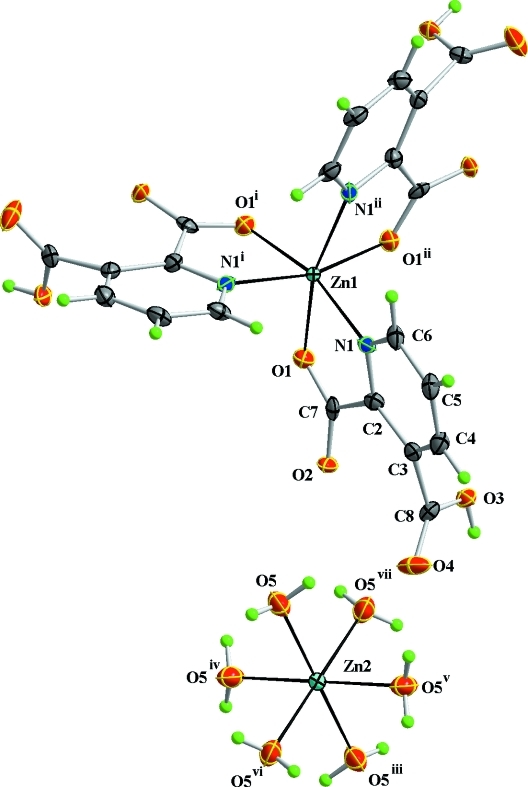
The molecular structure of the title compound, with the displacement ellipsoids drawn at 50% probability level.

**Fig. 2.**
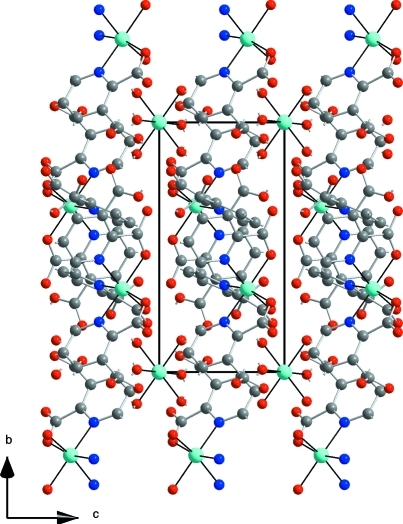
A view of along the a axis of the crystal packing in the title compounds. Layers perpendicular to c-directions are connected via hydrogen bonds.

## supplementary crystallographic information

### Comment

Metal-organic coordination complexes with one-, two- or three-dimensional structures have attracted attentions for their potential applications as photoelectric materials, catalysis, carriers, sensors, *etc*. (Prior & Rosseinsky, 2001; Swiegers & Malefetse, 2000). From a chemical point of view, L-lysine, a base, contains two amino groups and one carboxylic acid group, these amino groups often participate in hydrogen bonding and as a general base in catalysis. There are previously reported compounds containing pyridine-2,3-dicarboxylic acid, (py-2,3-dcH_2_), (Goher *et al.*, 1993; Yin & Liu 2009; Aghabozorg, Daneshvar *et al.*, 2007, Kang *et al.,* 2006, Li *et al.*, 2006).

The title compound consists of two [Zn(py-2,3-dcH)_3_]^¯^ anions and one Zn(H_2_O)_6_^2+^ cation. The anion is a six-coordinate complex located at a 3-fold crystallographic axis around Zn1 atom by three chelating (py-2,3-dcH)¯ ligands *via* one N and one O atom from carboxylate groups (Fig. 1). The cation is also six-coordinate with an octahedral geometry around Zn2 atom, located at a 3-bar axis. L-lysine, even it was included during the synthesis, is not part of this crystal structure and we were surprised that the product material contains Zn atoms in both form of cation and anion units. In anionic complex the three O—Zn1—N angles indicate that there is a distorted octahedral geometry around Zn1 atoms (for selected bond distances and angles see Table 1), but in cationic unit there are three O—Zn2—O angles exactly 180° as imposed by the crystallographic symmetry and good octahedral geometry environment around Zn2 atom (Table 1). The anionic Zn—O distances (Table 1) fall within the range of those found in related Zn complexes, 2.031- 2.117 Å (Aghabozorg, Daneshvar *et al.*, 2007; Aghabozorg, Sadr-khanlou *et al.*, 2007; Kang, *et al.*, 2006; Li, *et al.*, 2006;Yin, *et al.*, 2009).

There are three principal hydrogen bonds of O—H···O type (see Table 2) forming a complicated and extensive hydrogen bonding pattern. Graph-set analysis (Bernstein *et al.*, 1995) on the first level is indicating chains with symbols C^2^_2_(20) and C^2^_2_(16) for hydrogen bond with donor atoms O3 and O5, respectively, as well as ring *R*^2^_2_(20) for hydrogen bond with O3 as a donor. On second level graph-set, most important are ring *R*^1^_2_(6) and chains C^2^_2_(14) and C^2^_2_(16) between hexaaqua zinc cations, as well as rings *R*^3^_3_(15) formed between anions and cations.

There are π-π stacking interactions between anions proved by short distance *Cg4···Cg4* 3.828 (4)Å [*Cg*4 is centroid of N1/C2—C6 ring. Symmetry code: *1 - x,1 - y,1 - z*]. These π-π stacking interactions and O—H···O hydrogen bonds have important roles in stabilizing the structure. Crystal packing is depicted in Fig. 2.

### Experimental

The title compound was prepared by the reaction of pyridine-2,3-dicarboxylic acid (py-2,3-dcH_2_) (167 mg, 1 mmol), L-Lysine (L-Lys) (164 mg, 1 mmol) and with zinc(II) nitrate hexahydrate Zn(NO_3_)_2_. 6H_2_O (148.7 mg, 0.5 mmol), which were dissolved in distilled water (30 ml) as solvent in 2:2:1 molar ratio. The crystals were obtained by slow evaporation of solvent at room temperature.

### Refinement

Aromatic hydrogen atoms were refined isotropically with *U*_iso_(H) = 1.2*U*_eq_(C) and their positions were constrained to ideal geometry using an appropriate riding model, with C—H = 0.95. The carboxylate and water H atoms were located at the difference Fourier map and refined isotropically with *U*_iso_(H) = 1.5*U*_eq_(O), and restrained to ideal geometry with O—H distances 0.84 (2)Å and H···H distance 1.34 (2) Å for the water molecule.

### Crystal data

**Table:**

[Zn(H_2_O)_6_][Zn(C_7_H_4_NO_4_)_3_]_2_	*D*_x_ = 1.896 Mg m^−^^3^
*M**_r_* = 1300.88	Mo *K*α radiation, λ = 0.71073 Å
Trigonal, *P*3	Cell parameters from 1491 reflections
Hall symbol: -P 3	θ = 2.8–22.5°
*a* = 14.470 (4) Å	µ = 1.68 mm^−^^1^
*c* = 6.284 (2) Å	*T* = 153 K
*V* = 1139.4 (6) Å^3^	Block, colourless
*Z* = 1	1.20 × 0.44 × 0.42 mm
*F*(000) = 660	

### Data collection

**Table:**

Siemens SMART CCD area-detector diffractometer	1356 independent reflections
Radiation source: fine-focus sealed tube	1009 reflections with *I* > 2σ(*I*)
graphite	*R*_int_ = 0.154
ω scans	θ_max_ = 25.1°, θ_min_ = 2.8°
Absorption correction: multi-scan (*SADABS*; Sheldrick, 2003)	*h* = −17→17
*T*_min_ = 0.237, *T*_max_ = 0.538	*k* = −17→17
10776 measured reflections	*l* = −7→7

### Refinement

**Table:**

Refinement on *F*^2^	Secondary atom site location: difference Fourier map
Least-squares matrix: full	Hydrogen site location: inferred from neighbouring sites
*R*[*F*^2^ > 2σ(*F*^2^)] = 0.061	H atoms treated by a mixture of independent and constrained refinement
*wR*(*F*^2^) = 0.158	*w* = 1/[σ^2^(*F*_o_^2^) + (0.0993*P*)^2^ + 0.9035*P*] where *P* = (*F*_o_^2^ + 2*F*_c_^2^)/3
*S* = 1.00	(Δ/σ)_max_ < 0.001
1356 reflections	Δρ_max_ = 0.86 e Å^−^^3^
133 parameters	Δρ_min_ = −0.82 e Å^−^^3^
4 restraints	Extinction correction: *SHELXL97* (Sheldrick, 2008), Fc^*^=kFc[1+0.001xFc^2^λ^3^/sin(2θ)]^-1/4^
Primary atom site location: structure-invariant direct methods	Extinction coefficient: 0.035 (5)

### Special details

**Table:**

Geometry. All e.s.d.'s (except the e.s.d. in the dihedral angle between two l.s. planes) are estimated using the full covariance matrix. The cell e.s.d.'s are taken into account individually in the estimation of e.s.d.'s in distances, angles and torsion angles; correlations between e.s.d.'s in cell parameters are only used when they are defined by crystal symmetry. An approximate (isotropic) treatment of cell e.s.d.'s is used for estimating e.s.d.'s involving l.s. planes.
Refinement. Refinement of *F*^2^ against ALL reflections. The weighted *R*-factor *wR* and goodness of fit *S* are based on *F*^2^, conventional *R*-factors *R* are based on *F*, with *F* set to zero for negative *F*^2^. The threshold expression of *F*^2^ > σ(*F*^2^) is used only for calculating *R*-factors(gt) *etc*. and is not relevant to the choice of reflections for refinement. *R*-factors based on *F*^2^ are statistically about twice as large as those based on *F*, and *R*- factors based on ALL data will be even larger.

### Fractional atomic coordinates and isotropic or equivalent isotropic displacement parameters (Å^2^)

**Table:**

	*x*	*y*	*z*	*U*_iso_*/*U*_eq_	
Zn1	0.3333	0.6667	0.29441 (18)	0.0155 (4)	
O1	0.2510 (3)	0.5298 (3)	0.1090 (6)	0.0193 (9)	
O2	0.1958 (3)	0.3552 (3)	0.0954 (6)	0.0194 (10)	
O3	0.3623 (3)	0.2972 (3)	0.1662 (6)	0.0213 (10)	
H3	0.352 (5)	0.247 (4)	0.086 (9)	0.032*	
O4	0.2312 (4)	0.1865 (3)	0.3858 (7)	0.0322 (11)	
N1	0.3482 (4)	0.5491 (4)	0.4789 (7)	0.0161 (11)	
C2	0.3119 (4)	0.4565 (4)	0.3746 (8)	0.0155 (12)	
C3	0.3237 (4)	0.3729 (4)	0.4533 (9)	0.0167 (13)	
C4	0.3667 (5)	0.3851 (5)	0.6543 (10)	0.0216 (14)	
H4	0.3725	0.3283	0.7164	0.026*	
C5	0.4011 (4)	0.4781 (5)	0.7648 (9)	0.0200 (13)	
H5	0.4294	0.4863	0.9044	0.024*	
C6	0.3935 (4)	0.5601 (5)	0.6677 (9)	0.0183 (13)	
H6	0.4219	0.6267	0.7391	0.022*	
C7	0.2490 (4)	0.4467 (4)	0.1750 (9)	0.0168 (13)	
C8	0.2983 (5)	0.2751 (4)	0.3263 (9)	0.0187 (13)	
Zn2	0.0000	0.0000	0.0000	0.0226 (5)	
O5	0.0098 (3)	0.1271 (4)	0.1776 (7)	0.0305 (11)	
H51	−0.038 (3)	0.109 (5)	0.267 (8)	0.046*	
H52	0.069 (2)	0.161 (5)	0.238 (9)	0.046*	

### Atomic displacement parameters (Å^2^)

**Table:**

	*U*^11^	*U*^22^	*U*^33^	*U*^12^	*U*^13^	*U*^23^
Zn1	0.0124 (5)	0.0124 (5)	0.0217 (7)	0.0062 (2)	0.000	0.000
O1	0.017 (2)	0.016 (2)	0.024 (2)	0.0074 (18)	−0.0023 (17)	−0.0024 (17)
O2	0.016 (2)	0.011 (2)	0.031 (2)	0.0056 (18)	−0.0076 (17)	−0.0055 (17)
O3	0.019 (2)	0.016 (2)	0.029 (3)	0.0096 (19)	0.0048 (19)	−0.0047 (18)
O4	0.040 (3)	0.013 (2)	0.035 (3)	0.007 (2)	0.012 (2)	0.0014 (19)
N1	0.012 (2)	0.012 (2)	0.020 (3)	0.003 (2)	0.0024 (19)	−0.003 (2)
C2	0.013 (3)	0.013 (3)	0.018 (3)	0.005 (2)	0.001 (2)	0.002 (2)
C3	0.011 (3)	0.013 (3)	0.025 (3)	0.005 (2)	0.003 (2)	0.000 (2)
C4	0.021 (3)	0.021 (3)	0.027 (3)	0.014 (3)	0.003 (3)	0.001 (3)
C5	0.015 (3)	0.027 (3)	0.021 (3)	0.013 (3)	−0.002 (2)	−0.002 (3)
C6	0.014 (3)	0.025 (3)	0.018 (3)	0.012 (3)	−0.002 (2)	−0.005 (2)
C7	0.009 (3)	0.022 (3)	0.022 (3)	0.010 (3)	0.003 (2)	0.000 (3)
C8	0.020 (3)	0.015 (3)	0.026 (3)	0.012 (3)	−0.001 (3)	0.001 (2)
Zn2	0.0185 (6)	0.0185 (6)	0.0307 (11)	0.0092 (3)	0.000	0.000
O5	0.018 (2)	0.029 (3)	0.038 (3)	0.008 (2)	0.001 (2)	−0.004 (2)

### Geometric parameters (Å, °)

**Table:**

Zn1—O1	2.083 (4)	C3—C4	1.380 (8)
Zn1—N1	2.157 (5)	C3—C8	1.502 (8)
O1—C7	1.259 (7)	C4—C5	1.369 (8)
O2—C7	1.256 (7)	C4—H4	0.9500
O3—C8	1.294 (7)	C5—C6	1.386 (8)
O3—H3	0.83 (5)	C5—H5	0.9500
O4—C8	1.217 (7)	C6—H6	0.9500
N1—C6	1.327 (7)	Zn2—O5	2.095 (5)
N1—C2	1.340 (7)	O5—H51	0.82 (5)
C2—C3	1.393 (8)	O5—H52	0.83 (6)
C2—C7	1.514 (8)		
			
O1—Zn1—O1^i^	91.76 (15)	C5—C4—H4	119.7
O1—Zn1—N1^i^	165.35 (16)	C6—C5—C4	118.2 (5)
O1—Zn1—N1	77.62 (16)	C6—C5—H5	120.9
O1^i^—Zn1—N1	98.55 (15)	C4—C5—H5	120.9
N1^i^—Zn1—N1	93.83 (16)	N1—C6—C5	122.5 (5)
C7—O1—Zn1	116.9 (4)	N1—C6—H6	118.8
C8—O3—H3	117 (5)	C5—C6—H6	118.8
C6—N1—C2	118.7 (5)	O2—C7—O1	125.8 (5)
C6—N1—Zn1	128.6 (4)	O2—C7—C2	116.8 (5)
C2—N1—Zn1	112.5 (3)	O1—C7—C2	117.3 (5)
N1—C2—C3	122.5 (5)	O4—C8—O3	126.5 (5)
N1—C2—C7	114.5 (5)	O4—C8—C3	121.3 (5)
C3—C2—C7	122.8 (5)	O3—C8—C3	111.9 (5)
C4—C3—C2	117.2 (5)	O5—Zn2—O5^ii^	180.0
C4—C3—C8	119.2 (5)	O5—Zn2—O5^iii^	85.74 (18)
C2—C3—C8	123.5 (5)	Zn2—O5—H51	114 (5)
C3—C4—C5	120.6 (5)	Zn2—O5—H52	111 (5)
C3—C4—H4	119.7	H51—O5—H52	109 (3)
			
O1^iv^—Zn1—O1—C7	−172.0 (4)	C7—C2—C3—C4	−169.1 (5)
O1^i^—Zn1—O1—C7	96.2 (4)	N1—C2—C3—C8	−170.8 (5)
N1^iv^—Zn1—O1—C7	−94.3 (4)	C7—C2—C3—C8	14.7 (9)
N1^i^—Zn1—O1—C7	53.1 (8)	C2—C3—C4—C5	−3.3 (9)
N1—Zn1—O1—C7	−2.2 (4)	C8—C3—C4—C5	173.0 (5)
O1^iv^—Zn1—N1—C6	−132.6 (6)	C3—C4—C5—C6	−1.3 (9)
O1—Zn1—N1—C6	−176.9 (5)	C2—N1—C6—C5	−2.6 (8)
O1^i^—Zn1—N1—C6	93.2 (5)	Zn1—N1—C6—C5	−177.4 (4)
N1^iv^—Zn1—N1—C6	−79.0 (4)	C4—C5—C6—N1	4.5 (9)
N1^i^—Zn1—N1—C6	15.1 (5)	Zn1—O1—C7—O2	173.2 (4)
O1^iv^—Zn1—N1—C2	52.4 (8)	Zn1—O1—C7—C2	−3.5 (6)
O1—Zn1—N1—C2	8.0 (4)	N1—C2—C7—O2	−166.2 (5)
O1^i^—Zn1—N1—C2	−81.9 (4)	C3—C2—C7—O2	8.7 (8)
N1^iv^—Zn1—N1—C2	105.9 (4)	N1—C2—C7—O1	10.8 (7)
N1^i^—Zn1—N1—C2	−159.9 (4)	C3—C2—C7—O1	−174.3 (5)
C6—N1—C2—C3	−2.5 (8)	C4—C3—C8—O4	63.8 (8)
Zn1—N1—C2—C3	173.1 (4)	C2—C3—C8—O4	−120.1 (7)
C6—N1—C2—C7	172.4 (5)	C4—C3—C8—O3	−110.0 (6)
Zn1—N1—C2—C7	−12.0 (6)	C2—C3—C8—O3	66.1 (7)
N1—C2—C3—C4	5.4 (8)		

Symmetry codes: (i) −*y*+1, *x*−*y*+1, *z*; (ii) −*x*, −*y*, −*z*; (iii) *x*−*y*, *x*, −*z*; (iv) −*x*+*y*, −*x*+1, *z*.

### Hydrogen-bond geometry (Å, °)

**Table:**

*D*—H···*A*	*D*—H	H···*A*	*D*···*A*	*D*—H···*A*
O3—H3···O2^v^	0.83 (5)	1.72 (2)	2.547 (5)	169 (7)
O5—H52···O4	0.83 (5)	2.38 (4)	3.157 (7)	156 (7)
O5—H51···O4^vi^	0.82 (6)	2.02 (3)	2.795 (6)	158 (6)

Symmetry codes: (v) *y*, −*x*+*y*, −*z*; (vi) −*y*, *x*−*y*, *z*.
